# Comparative Pharmacokinetic Study of 5 Active Ingredients after Oral Administration of Zuogui Pill in Osteoporotic Rats with Different Syndrome Types

**DOI:** 10.1155/2023/1473878

**Published:** 2023-02-25

**Authors:** Jiawei Qiu, Yaoyao Zhu, Jing Xing, Ling Wang, Jianhua Zhang, Hua Yin

**Affiliations:** ^1^School of Pharmaceutical Sciences, Zhejiang Chinese Medical University, Hangzhou 311402, China; ^2^Standardization of Chinese Medicine Research Laboratory, Zhejiang Chinese Medical University, Hangzhou 311402, China; ^3^The First Affiliated Hospital of Zhejiang Chinese Medical University, Hangzhou 310006, China

## Abstract

Zuogui Pill is a kidney-yin-tonifying formula in traditional Chinese medicine that is widely used to manage osteoporosis with kidney-yin-deficiency in China. Herein, an efficient and accurate high-performance liquid chromatography-tandem mass spectrometry method was developed to determine the concentrations of 5 bioactive compounds in rat plasma following oral administration of Zuogui Pill. Because drug absorption and distribution differ under physiological and pathological conditions, the established method was used to quantify blood components and dynamic change in osteoporotic rats with different syndrome types. Moreover, integrated pharmacokinetic study was conducted to describe the overall pharmacokinetic characteristics of traditional Chinese medicine. The results showed that the absorption, distribution, and metabolism of Zuogui Pill varied widely under different states. The bioavailability of most active components showed significant advantages in osteoporotic rats with kidney-yin-deficiency, which corresponds to the opinion that Zuogui Pill has the effect of nourishing kidney-yin. It is hoped that this finding could interpret the pharmacodynamic substances and mechanism of Zuogui Pill in the treatment of osteoporosis with kidney-yin-deficiency.

## 1. Introduction

Osteoporosis is a progressive bone remolding disease characterized by osteopenia and bone structure deterioration. The essence of osteoporosis is an imbalance between bone formation and bone resorption, which can cause skeletal fragility, increased fracture risk, and post-fracture mortality [1]. Recent evidence shows that the incidence of osteoporosis has been increasing year by year. Global osteoporosis-induced hip fracture patients are expected to increase by 550% by 2050 [2, 3]. Hormone replacement therapy (HRT) is currently proven to be effective in preventing bone loss and lowering the risk of fractures in osteoporotic patients [4, 5]. Nonetheless, long-term HRT has been linked to an increased risk of a variety of diseases [6, 7]. Concerns about the adverse effects of HRT have heightened interest in developing alternative strategies for osteoporosis treatment, and traditional Chinese medicine (TCM) has attracted considerable interest due to its superior curative effect and lower side effects. The TCM theory points out that the prevalence of osteoporosis is basically kidney deficiency; clinically, it mainly manifests as kidney-yang-deficiency and kidney-yin-deficiency syndrome [8–10]. As a result, kidney-tonifying is a common and traditional strategy for treating bone diseases such as osteoporosis with TCM herbal formulas that have kidney-tonifying activity.

**Figure 1:**
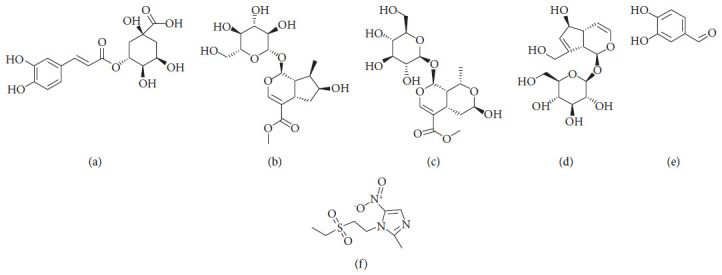
Chemical structures of chlorogenic acid (a), loganin (b), morroniside (c), aucubin (d), protocatechuic aldehyde (e), and tinidazole (IS) (f).

Zuogui Pill is a classic kidney-yin-tonifying formula which was developed by a famous TCM doctor Jingyue Zhang during the Ming dynasty (1563–1640 AD). The formula consists of Rehmanniae Radix Praeparata, Dioscoreae Rhizoma, Lycii Fructus, Corni Fructus, Cyathulae Radix, Cuscutae Semen, Cervi Cornus Colla, and Testudinis Carapacis et Plastri Colla. The majority of TCM in this formula have similar and synergistic kidney-yin-tonifying functions and thus have the potential to benefit bone health [11, 12]. Many bioactive ingredients in Zuogui Pill, including aucubin, loganin, chlorogenic acid, and morroniside can promote osteoblast differentiation, reduce calcitonin levels, and inhibit osteoblast apoptosis and osteoclast proliferation [13–17]. Meanwhile, protocatechuic aldehyde has been shown to be effective in the prevention and treatment of osteoporosis [18, 19]. Therefore, we selected the 5 bioactive components of Zuogui Pill (Figure 1) for further study.

Mounting evidence shows that differences in body conditions can influence drug absorption and distribution [20, 21]. From the perspectives of serum pharmacochemistry research ideas, only the components that can be absorbed are important for clinical effects [22]. Thus, studying pharmacokinetic properties in rats under pathological and normal conditions may reveal the mechanism of Zuogui Pill in the treatment of osteoporosis. Up to now, the methods for qualitative and quantitative detection of chlorogenic acid, loganin, and morroniside in Zuogui Pill have been established [23, 24]. There is no pharmacokinetic study on the active ingredients of Zuogui Pill in rat plasma. In this study, we developed an efficient and rapid HPLC-MS/MS method for detecting 5 components in plasma. Following that, the developed method was successfully used to compare the pharmacokinetic characteristics of Zuogui Pill after oral administration in normal rats, osteoporotic rats, and osteoporotic rats with kidney-yang-deficiency and kidney-yin-deficiency syndrome. Due to the complex composition of TCM compound, any single component poses limitations in describing the overall pharmacokinetic characteristics of TCM. Consequently, a study of integrated pharmacokinetics in each syndrome was conducted for systematic evaluation [25].

## 2. Materials and Methods

### 2.1. Chemicals and Reagents

Rehmanniae Radix Praeparata, Dioscoreae Rhizoma, Lycii Fructus, Corni Fructus, Cyathulae Radix, Cuscutae Semen, Cervi Cornus Colla, and Testudinis Carapacis were purchased from Huadong Pharmaceutical (Hangzhou, China) and confirmed by Professor Rusong Zhang (School of Pharmaceutical Science, Zhejiang Chinese Medical University).

Aucubin, loganin, protocatechuic aldehyde, chlorogenic acid, and tinidazole (internal standard, IS) (purity > 98%) were obtained from the National Institutes for Food and Drug Control (Beijing, China). Morroniside (purity > 98%) was procured from Beijing Yanxinlv Biological Technology (Beijing, China). Methanol, acetonitrile, ammonium formate, and formic acid (HPLC-grade) were obtained from Merck (Darmstadt, Germany). Hydrocortisone injections were provided by Jinyao Amino Acid Co. Ltd. (Tianjin, China). Thyroxine tablets were supplied by Great Wall Pharmaceutical Co. Ltd. (Shanghai, China). Ultrapure water was purchased from Wahaha Co. Ltd. (Hangzhou, China).

### 2.2. Preparation of Zuogui Pill Extract

The Zuogui Pill extract was prepared using a previously described method [26]. The raw materials, which included Rehmanniae Radix Praeparata (24 g), Dioscoreae Rhizoma (12 g), Lycii Fructus (12 g), Corni Fructus (12 g), Cyathulae Radix (9 g), Cuscutae Semen (12 g), Cervi Cornus Colla (12 g), and Testudinis Carapacis (12 g), were sliced and soaked in a 10-fold volume of 50% ethanol for 60 min. After that, a 30 min ultrasonic extraction was performed twice at 45°C. The extract solution was filtered and condensed to a 4 g/ml concentration.

### 2.3. Animals and Establishment of Osteoporosis Models

Female Sprague-Dawley rats (180–220 g) were purchased from Shanghai Laboratory Animal Centre (Shanghai, China). The rats were maintained in a standard environment (22 ± 1°C, 60 ± 10% relative humidity, and a 12 h light/dark cycle) for one week to acclimate and fed water and food ad libitum. Twenty-four rats were divided randomly into three groups and treated through ovary enucleation to establish the model of osteoporosis [27]. Ten weeks after operation, osteoporosis models of kidney-yang-deficiency and kidney-yin-deficiency were established by intramuscular injection of hydrocortisone (25 mg/kg) and intragastric administration of thyroxine suspension (160 mg/kg) for 10 days, respectively. The femur of two rats from each group was then stained with hematoxylin and eosin (H&E) to assess histopathological change. Also, the validation of osteoporosis kidney-yang-deficiency and kidney-yin-deficiency models was judged by detecting the level of cyclic adenosine monophosphate (cAMP) and cyclic guanosine monophosphate (cGMP) in rat plasma [28]. Laboratory Animal Management and Ethics Committee of Zhejiang Chinese Medical University approved all animal treatments (ZSLL-2014-48).

### 2.4. Preparation of Standards and Quality Controls

The standards of 5 active compounds and IS were weighed and solubilized in methanol to 1.0 mg/mL. To make working solutions, stock solutions were diluted with methanol, and the IS working solutions were diluted in a methanol : acetonitrile ratio of 1 : 1 at a final concentration of 50 ng/mL.

To obtain calibration standards, 10 *μ*L working solutions were added in 90 *μ*L blank plasma. The final concentrations were 1–1000 ng/mL for chlorogenic acid, 20–20000 ng/mL for loganin, 4–4000 ng/mL for morroniside, 0.5–500 ng/mL for aucubin, and 0.05–50 ng/mL for protocatechuic aldehyde.

Quality control (QC) samples were prepared at the following concentrations: 2, 40, and 800 ng/mL for chlorogenic acid, 40, 800, and 16000 ng/mL for loganin, 8, 160, and 3200 ng/mL for morroniside, 1, 20, and 400 ng/mL for aucubin, and 0.1, 2, and 40 ng/mL for protocatechuic aldehyde.

### 2.5. Sample Preparation

One hundred microliters of plasma was mixed with 100 *μ*L of the IS solution, followed by 700 *μ*L of methanol:acetonitrile (1 : 1). After 2 min of vortex mixing, the mixture was spun for 15 min at 8000 rpm and 4°C. The supernatants were collected and dried in a high-purity N_2_ environment. The residues were redissolved in 100 *μ*L methanol and centrifuged for 15 min at 13000 rpm and 4°C. Finally, an aliquot of 10 *μ*L supernatants was used to conduct the analysis.

### 2.6. Instrument and Analytical Conditions

An Agilent 1290 HPLC system (Agilent, California, USA) was used in conjunction with an AB Sciex Q-TRAP 5500 MS/MS system (AB Sciex, Foster City, USA) equipped with an ESI source to measure the 5 components.

The LC separation was performed on a Waters Atlantis C18 column (3.0 mm × 150 mm, 3 *μ*m) with a mobile phase of 5 mmol/L ammonium formate in 0.05% formic acid aqueous solution (A) and acetonitrile (B) flowing at a rate of 0.3 mL/min. The gradient elution program was performed as follows: 0-1 min, 90% A; 1–5 min, 90–20% A; 5–10 min, 20% A; 10–10.1 min, 20–90% A; and 10.1–12 min, 90% A. The column temperature was 20°C.

The mass spectrometer was set to detect in a negative mode under the following conditions: ion spray voltage, 4500 V; curtain gas, 40 psi; source temperature, 650°C; and collision gas, medium. The analytes were quantified using multiple reaction monitoring, and the optimized parameters are listed in Table 1.

### 2.7. Method Validation

The methods were validated in accordance with US Food and Drug Administration guidelines for bioanalytical method validation [29].

#### 2.7.1. Specificity

Chromatograms of blank biological samples from six rats, plasma samples containing standards, and plasma samples after administration of Zuogui Pill extract were compared to assess selectivity.

#### 2.7.2. Linearity and Sensitivity

Calibration curves were constructed by drawing peak area ratios of 5 compounds to IS against nominal concentrations using least squares regression (weighted factor: 1/*x*^2^). The lower limit of quantification (LLOQ) was defined as the lowest level on calibration curves with a signal-to-noise ratio (S/N) = 10 used to demonstrate method sensitivity.

#### 2.7.3. Accuracy and Precision

Six batches of QC samples at three levels and LLOQ were used to determine accuracy and precision. To determine intra-day accuracy and precision, samples were analysed on a single assay day, while inter-day accuracy and precision were determined over three consecutive days.

#### 2.7.4. Extraction Recovery and Matrix Effect

To determine extraction recovery, peak areas of analytes in plasma samples were compared to post-extraction plasma containing pure analyte standards at corresponding concentrations. The matrix effect was calculated by comparing the peak areas of analytes added to blank plasma after extraction to those of standards liquefied in methanol at comparable concentrations.

#### 2.7.5. Stability

QC samples at three levels using five replicates were used to evaluate the stability under the following conditions: 4 h at room temperature (25°C), 24 h in autosampler (4°C), and three freeze-thaw cycles (−20°C to 25°C).

### 2.8. Pharmacokinetic Study and Statistical Analysis

Four groups of rats were orally administered with Zuogui Pill extract at a dose of 1.5 mL/100 g. Then, 0.3 mL blood samples were collected from orbital veins at 0, 0.08, 0.17, 0.25, 0.5, 1, 2, 4, 6, 8, 12, and 24 h after administration, placed in an anticoagulant tube, and centrifuged for 15 min at 5000 rpm and 4°C. The plasma was prepared and stored at −20°C until analysis.

Phoenix WinNonlin 6.4 software was used to estimate pharmacokinetic parameters using noncompartmental analysis. Furthermore, SPSS 26 software was used to compute comparisons of the pharmacokinetic data among the four groups according to a one-way ANOVA. The results are presented as mean ± SD.

### 2.9. Integrated Pharmacokinetic Study

Integrated pharmacokinetic study was conducted based on the area under the concentration-time curve from zero to infinity (AUC_0-∞_) of each component [30, 31]. The ratio of AUC_0-∞_ of 5 components to total AUC_0-∞_ was defined as the weight coefficient (*ω*) in the integrated concentration using equations (1) and (2). Formula (3) was used for obtaining the integrated concentration of the 5 components, where *C*_1_–*C*_5_ represent the concentration of the five components, and the integrated pharmacokinetic parameters were further calculated.

(1) $$\begin{array}{c} \omega_{j}=\frac{{AUC}_{0–\infty \left(j\right)}}{\sum_{1}^{5}{AUC}_{0–\infty }}\text{,} \end{array}$$

(2) $$\begin{array}{c} \overset{5}{\underset{1}{\sum }}{AUC}_{0–\infty }={AUC}_{0–\infty \left(1\right)}+{AUC}_{0–\infty \left(2\right)}+{AUC}_{0–\infty \left(3\right)}+{AUC}_{0–\infty \left(4\right)}+{AUC}_{0–\infty \left(5\right)}\text{,} \end{array}$$

(3) $$\begin{array}{c} CT=\omega_{1}\times C_{1}+\omega_{2}\times C_{2}+\omega_{3}\times C_{3}+\omega_{4}\times C_{4}+\omega_{5}\times C_{5}\text{.} \end{array}$$

## 3. Results and Discussion

### 3.1. Evaluation of Osteoporosis Models

**Figure 2:**
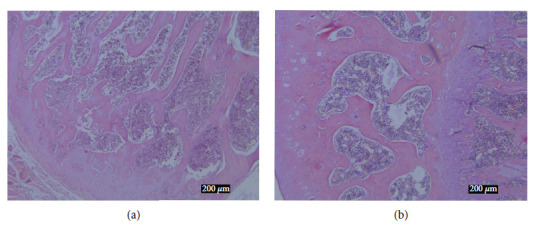
Pathological section of the femur in normal (a) and osteoporotic (b) rats.

H&E staining revealed that the subchondral bone and trabecula were intact and arranged regularly in the normal rats. Osteocytes in trabecular bone were clearly visible, and the nuclei were centrally located and large. The bone marrow was rich in hematopoietic cells, with relatively few adipocytes in the medullary cavity and normal morphology, while the trabecular bone area and the number of hematopoietic cells were significantly reduced in the osteoporosis group. In addition, the volume of adipocytes increased and even fused into vesicles (Figure 2), both of which confirmed the successful establishment of the osteoporosis model.

The contents of cAMP and cGMP in rat plasma are covered in Table 2. After modelling, the plasma content of cAMP in the osteoporotic rats with kidney-yang-deficiency significantly decreased (*P* < 0.01), while that of cGMP increased (*P* < 0.01) compared with the osteoporosis group. Also, the levels of plasma cAMP and cGMP showed the opposite trend in kidney-yin-deficiency rats. The increase or decrease of the cAMP/cGMP ratio indicated that the two types of osteoporosis models were successfully established.

### 3.2. Operational Condition Optimization

Considering the low drug concentration in plasma due to its first-pass effect, QTOF-MS was used for analysis in the pre-experiment. However, the sensitivity of QTOF-MS cannot meet the quantitative requirements; therefore, Q-TRAP MS was employed for detection in the end.

Different types of columns were tested, including Agilent ZORJBAX Extend *C*_18_ column (4.6 mm × 150 mm, 5 *μ*m), Agilent ZORJBAX SB *C*_18_ column (4.6 mm × 150 mm, 5 *μ*m) and Waters Atlantis *C*_18_ column (3.0 mm × 150 mm, 3 *μ*m), and it turned out that Waters Atlantis *C*_18_ column (3.0 mm × 150 mm, 3 *μ*m) could present a better peak shape and isolation. Acetonitrile, methanol, and several volatile acid-base additives in the aqueous phase such as formic acid, ammonia, ammonium formate, and ammonium acetate were evaluated to optimize the chromatography conditions. Finally, acetonitrile and 5 mmol/L ammonium formate in 0.05% formic acid aqueous solution were chosen as the mobile-phase system.

**Figure 3:**
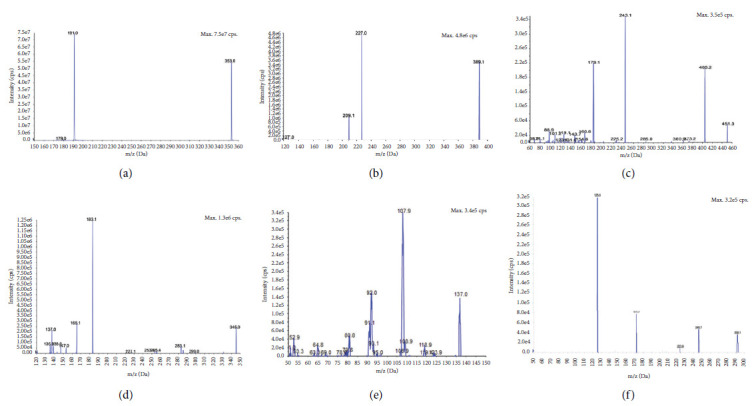
Mass spectrograms of chlorogenic acid (a), loganin (b), morroniside (c), aucubin (d), protocatechuic aldehyde (e), and tinidazole (IS) (f).

Because the response value of analytes to be measured was higher in the negative ion ionization state than in the positive mode, the former was used for the assay.  Figure 3 depicts the ion mass spectrums.

### 3.3. Concentrations of the 5 Compounds in Zuogui Pill

The contents of 5 analytes were evaluated according to the method described in Section 2.6. The contents of chlorogenic acid, loganin, morroniside, aucubin, and protocatechuic aldehyde in Zuogui Pill were 33.2, 479, 411, 16.2, and 5.61 *μ*g/mL, respectively.

### 3.4. Method Validation

#### 3.4.1. Specificity

**Figure 4:**
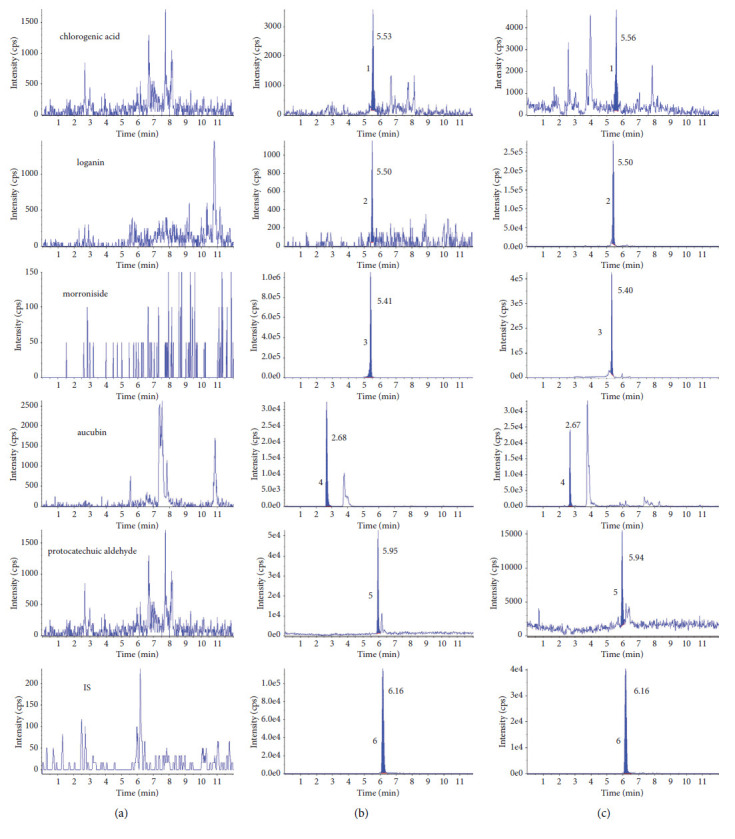
Multiple reaction monitoring chromatograms of each analyte and IS: (a) blank plasma; (b) blank plasma spiked with analytes and IS; (c) plasma sample obtained 30 min after oral administration of Zuogui Pill.

Figure 4 shows chromatograms of different plasma, including blank plasma, blank plasma with standards, and plasma sample from a rat 30 min after oral administration. The results demonstrated that endogenous plasma components did not influence the test substance or internal standard. Also, the IS response in the blank sample was no more than 5% of the average IS responses of the calibrators and QCs.

#### 3.4.2. Linearity and Sensitivity

Analyte calibration curves were linear over their respective ranges, with correlation coefficients (*R*) greater than 0.9990 for all sequences. The data in Table 3 demonstrated that the linearity and LLOQ were suitable for assay with acceptable precision and accuracy.

#### 3.4.3. Accuracy and Precision

Table 4 shows the results of the inter- and intra-day accuracy and precision of the 5 components. The precision was less than 15%, while the accuracy was 2.3% to 18.6% for intra-day assay and 2.5% to 18.0% for inter-day assay. All the data showed that the method was reliable and accurate.

#### 3.4.4. Extraction Recovery and Matrix Effect

The extraction recovery of the analytes at different selected levels ranged from 65.0% to 91.9%, and the average matrix effect at the same concentrations ranged from 76.3% to 94.4% (Table 5). The absence of matrix components in plasma results in a striking variation in the mass spectrum response to analytes and IS, which is consistent with the analytical requirements of the biological sample.

#### 3.4.5. Stability

The stability analysis of the 5 compounds revealed no obvious degradation of components detected in rat plasma under the above storage conditions with RSD below 15% (Table 6).

### 3.5. Pharmacokinetic Analysis

**Figure 5:**
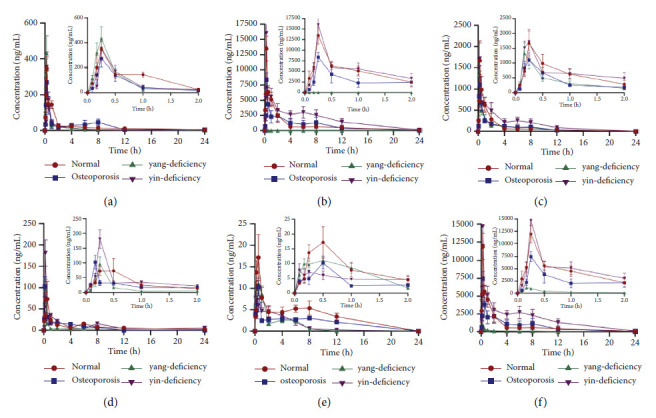
Drug concentration-time profiles of chlorogenic acid (a), loganin (b), morroniside (c), aucubin (d), and protocatechuic aldehyde (e) and integrated blood concentration-time comparison chart (f) (*n* = 6).

The validated method was used for pharmacokinetic assays of the 5 active compounds in plasma following oral administration of Zuogui Pill.  Figure 5shows the mean plasma concentrations versus time profiles of 5 compounds and integration under various syndrome types. Table 7 displays the pharmacokinetic variables of the 5 compounds as well as the integrated pharmacokinetic variables.

Although the dosage of Zuogui Pill in each group was the same, the pharmacokinetic parameters of the four groups were dramatically different. The elimination half-life (*t*_1/2_) of chlorogenic acid, loganin, morroniside, and aucubin in the osteoporosis group was shorter than that in the normal group, indicating that they were eliminated faster in the osteoporosis state. Clearance (CL) for the 5 compounds was increased in osteoporotic rats. All the components were absorbed and reached their peak plasma concentration in 1 h. Aside from aucubin, the maximum plasma concentration (*C*_max_) of the remaining four components was higher in normal rats than in osteoporotic rats. Furthermore, in the osteoporosis group, the AUC_0–*t*_ and AUC_0–∞_ of all ingredients were reduced, particularly in protocatechuic aldehyde (*P* < 0.05). By comparing the pharmacokinetic parameters in normal and osteoporosis groups, we found that the bioavailability of the 5 components in normal rats was higher than that in osteoporotic rats, while the removal rates of the 5 components in normal rats were lower.

The clearance of all analytes was statistically increased (*P* < 0.05) in kidney-yang-deficiency rats, while the AUC_0–*t*_ and AUC_0–∞_ of chlorogenic acid, loganin, aucubin, and protocatechuic aldehyde were significantly decreased (*P* < 0.01), suggesting that the above components were poorly absorbed and rapidly metabolized in this state. Except for protocatechuic aldehyde, *C*_max_ of other compounds increased remarkably (*P* < 0.05) in the kidney-yin-deficiency group when compared with the osteoporosis group. Increases in the value of mean residence time (MRT), AUC_0–*t*_, and AUC_0–∞_ of loganin, morroniside, and aucubin were observed (*P* < 0.05) in the kidney-yin-deficiency group. The results demonstrated that the three components had better absorption and a long duration, which may lead to a significant accumulation of active components in vivo. Thus, loganin, morroniside, and aucubin are likely to be the medicinal compositions in the treatment of osteoporosis with kidney-yin-deficiency in Zuogui Pill extract as reported in the literature [32].

The above changes in pharmacokinetic behaviours could be due to the fact that different types of osteoporosis potentially influence the absorption, distribution, and metabolism of drugs. One of the factors influencing the rate and quantity of drug absorption in osteoporosis is the change in gut microbiota and metabolite [33, 34]. The phenomenon that all the components showed double or multiple peaks in the mean plasma concentration-time profiles could be attributed to Zuogui Pill reabsorption in plasma and enterohepatic circulation following oral administration [35, 36].

### 3.6. Integrated Pharmacokinetic Analysis

TCM compounds are formulated in accordance with the composition theory of a TCM prescription, which contains complex chemical components that potentially interact with one another to influence their pharmacokinetic behaviours [37]. The results of our assay revealed significant differences in the pharmacokinetic parameters of 5 compounds, demonstrating that any single ingredient poses limitations in describing pharmacokinetic behaviour in rats. The integrated pharmacokinetic parameters, on the other hand, were more practical and reasonable. According to Figure 5 and Table 6, it was obvious that the bioavailability and effective drug duration of integrated ingredient showed significant advantages in the state of kidney-yin-deficiency by comparison with other groups, which was consistent with the TCM theory that Zuogui Pill has the effect of nourishing kidney-yin [38].

## 4. Conclusions

The presently developed HPLC-MS/MS method is efficient and effective in detecting the 5 analytes in plasma. Therefore, it was used to compare the pharmacokinetics of 5 components in different syndrome types of osteoporosis. The evaluation of the pharmacokinetic behaviours of the research objects may aid in better understanding the mechanism of the pharmacological action and provide references on the clinical application of Zuogui Pill.

## Figures and Tables

**Table 1:** Mass spectrometric parameters of 5 analytes and IS.

Analytes	*Q*1 (m/z)	*Q*3 (m/z)	DP (V)	EP (V)	CE (eV)	CXP (V)
Chlorogenic acid	353.1	191.0	−68	−10	−24	−17
Loganin	389.1	227.0	−78	−7	−36	−15
Morroniside	451.1	243.0	−50	−7	−23	−22
Aucubin	345.0	183.0	−50	−6	−21	−15
Protocatechuic aldehyde	137.0	107.9	−37	−10	−30	−10
IS	292.0	126.1	−52	−11	−17	−12

**Table 2:** Plasma concentrations of cAMP and cGMP in rats (*n* = 6).

Groups	cAMP (nmol/L)	cGMP (nmol/L)	cAMP/cGMP
Normal	101.7 ± 5.971	25.62 ± 3.176	3.901 ± 0.327
Osteoporosis	100.4 ± 3.302	26.46 ± 1.498	3.805 ± 0.233
Kidney-yang-deficiency	85.30 ± 5.061^*∗∗*^	35.84 ± 3.170^*∗∗*^	2.393 ± 0.234^*∗∗*^
Kidney-yin-deficiency	119.1 ± 9.349^*∗*^	23.20 ± 1.357^*∗*^	5.129 ± 0.509^*∗∗*^

^*∗*^ *p* < 0.05 and ^*∗∗*^*p* < 0.01, according to *t*-test, versus osteoporosis rats.

**Table 3:** Calibration curves, correlation coefficients, linear ranges, and LLOQs of 5 compounds.

Compounds	Calibration curves	Correlation coefficients (*r*)	Linear range (ng/mL)	LLOQ (ng/mL)
Chlorogenic acid	*y* = 0.0226*x* − 0.0985	0.9997	1–1000	1
Loganin	*y* = 0.0023*x* + 0.0297	0.9996	20–20000	20
Morroniside	*y* = 0.0114*x* + 0.1404	0.9993	4–4000	4
Aucubin	*y* = 0.0016*x* − 0.0175	0.9990	0.5–500	0.5
Protocatechuic aldehyde	*y* = 0.1696*x* + 2.2261	0.9992	0.05–50	0.05

**Table 4:** Accuracy and precision of 5 analytes in rat plasma (*n* = 6).

Analytes	Concentration (ng/mL)	Accuracy (%)		Precision (%)	
		Intra-day	Inter-day	Intra-day	Inter-day
Chlorogenic acid	1	18.6	18.0	12.4	9.5
	2	12.1	14.3	10.0	6.1
	40	10.2	11.0	10.3	7.6
	800	6.7	8.1	3.9	8.2

Loganin	20	17.4	14.2	10.6	13.7
	40	13.8	12.1	7.1	10.9
	800	7.5	5.6	6.4	5.8
	16000	2.3	2.5	3.9	4.1

Morroniside	4	13.3	16.6	10.6	12.0
	8	11.1	9.4	8.7	8.7
	160	6.6	9.2	7.3	2.9
	3200	3.4	8.6	5.9	3.1

Aucubin	0.5	13.0	14.7	10.4	10.5
	1	12.5	9.0	7.5	8.0
	20	3.7	7.1	5.8	5.3
	400	4.0	5.3	3.7	4.9

Protocatechuic aldehyde	0.05	11.4	14.6	10.2	13.8
	0.1	8.0	13.2	9.6	9.1
	2	7.7	13.2	7.4	8.4
	40	6.2	8.8	4.4	4.9

**Table 5:** Recover and matrix effect of 5 analytes in rat plasma (*n* = 6).

Analytes	Concentration (ng/mL)	Recovery (%)		Matrix effect (%)	
		Mean	RSD	Mean	RSD
Chlorogenic acid	2	65.0	8.5	85.3	6.1
	40	88.2	4.6	90.7	4.4
	800	78.0	10.3	86.8	4.7

Loganin	40	81.3	3.3	76.3	14.9
	800	87.6	9.7	93.7	5.9
	16000	91.9	7.0	86.5	3.7

Morroniside	8	80.3	1.8	92.0	8.3
	160	82.2	6.6	76.7	5.4
	3200	84.1	10.7	85.5	1.9

Aucubin	1	76.7	4.3	80.7	6.1
	20	77.7	3.3	94.4	14.4
	400	88.0	2.6	91.8	8.9

Protocatechuic aldehyde	0.1	85.4	2.7	81.5	6.1
	2	85.8	9.6	79.4	6.0
	40	85.9	8.9	91.5	4.5

**Table 6:** Stability of 5 analytes in rat plasma under various conditions (*n* = 5).

Analytes	Concentration (ng/mL)	Room temperature for 4 h (%)		Autosampler at 4°C for 24 h (%)		Three freeze-thaw cycles (%)	
		Mean	RSD	Mean	RSD	Mean	RSD
Chlorogenic acid	2	86.5	6.0	85.1	4.6	89.1	5.1
	40	85.9	2.2	93.7	3.8	89.3	7.7
	800	85.7	4.2	87.3	6.2	80.9	10.9

Loganin	40	88.3	9.5	94.6	5.1	86.4	12.4
	800	87.4	6.1	85.8	7.0	89.5	6.9
	16000	85.6	11.3	84.4	3.9	92.4	3.3

Morroniside	8	90.8	9.1	89.6	5.8	93.0	2.7
	160	86.1	7.5	87.6	4.6	91.5	6.3
	3200	98.3	3.4	95.0	2.1	89.8	7.7

Aucubin	1	89.8	2.8	88.6	4.7	84.2	9.0
	20	91.3	8.0	92.5	7.4	86.9	8.3
	400	92.8	6.6	90.2	5.8	89.1	6.1

Protocatechuic aldehyde	0.1	86.8	2.5	88.4	8.9	90.8	6.5
	2	90.9	3.3	89.3	6.5	83.4	5.7
	40	87.4	6.0	85.9	3.1	89.5	4.4

**Table 7:** Pharmacokinetic parameters of the 5 compounds in rats after oral administration of Zuogui Pill extract and integrated pharmacokinetic parameters (*n* = 6).

Compounds	Groups	*t*_1/2_(h)	*T*_max_(h)	*C*_max_(ng/mL)	AUC_0–*t*_(h^*∗*^ng/mL)	AUC_0–∞_(h^*∗*^ng/mL)	CL_F_obs (mL/h/kg)	MRT_0–t_(h)	MRT_0–∞_(h)
Chlorogenic acid	Normal	8.17 ± 1.79	0.25 ± 0.00	346.14 ± 63.99	497.98 ± 34.72^*∗*^	546.26 ± 38.80	914.49 ± 64.88	5.46 ± 0.48^*∗*^	8.17 ± 1.46
	Osteoporosis	6.38 ± 1.96	0.26 ± 0.02	279.27 ± 56.34	435.76 ± 59.78	489.67 ± 77.69	1036.09 ± 154.41	6.78 ± 0.68	9.72 ± 2.61
	Yang-deficiency	5.41 ± 1.84	0.25 ± 0.00	430.29 ± 98.55^*∗∗*^	263.13 ± 40.25^*∗∗*^	284.31 ± 33.34^*∗∗*^	1767.90 ± 187.68^*∗∗*^	4.52 ± 1.46	6.56 ± 2.64^*∗*^
	Yin-deficiency	6.61 ± 3.84	0.25 ± 0.00	353.83 ± 83.80	218.55 ± 23.29^*∗∗*^	225.89 ± 25.03^*∗∗*^	2223.36 ± 229.72^*∗∗*^	3.03 ± 0.38^*∗∗*^	4.10 ± 1.02^*∗∗*^

Loganin	Normal	4.51 ± 0.45^*∗∗*^	0.25 ± 0.00	13508.61 ± 2029.85^*∗∗*^	20134.29 ± 3958.79	20523.97 ± 3947.05	361.54 ± 73.02	4.58 ± 0.47	5.10 ± 0.41
	Osteoporosis	2.74 ± 0.96	0.25 ± 0.00	8381.77 ± 1123.47	17912.12 ± 6898.16	17998.56 ± 6945.75	460.13 ± 196.92	4.91 ± 1.29	5.02 ± 1.34
	Yang-deficiency	5.62 ± 1.63^*∗∗*^	0.21 ± 0.05	14.72 ± 1.38^*∗∗*^	27.46 ± 4.83^*∗∗*^	28.61 ± 4.91^*∗∗*^	257023.69 ± 42243.40^*∗∗*^	5.50 ± 0.67	6.62 ± 0.49
	Yin-deficiency	3.75 ± 0.70	0.25 ± 0.00	16025.83 ± 2787.58^*∗∗*^	41028.61 ± 6356.26^*∗∗*^	41727.10 ± 6203.34^*∗∗*^	176.22 ± 32.93	6.51 ± 0.91^*∗∗*^	6.92 ± 0.98

Morroniside	Normal	3.66 ± 1.18 ^*∗*^	0.29 ± 0.10	1719.66 ± 410.02^*∗∗*^	2151.00 ± 498.88	2171.33 ± 509.45	2966.14 ± 684.64^*∗*^	3.41 ± 0.33^*∗∗*^	3.66 ± 0.48^*∗*^
	Osteoporosis	2.40 ± 0.51	0.26 ± 0.02	1109.11 ± 163.31	1724.77 ± 149.05	1741.55 ± 136.08	3553.71 ± 268.04	4.37 ± 0.43	4.51 ± 0.38
	Yang-deficiency	3.83 ± 0.64^*∗∗*^	0.19 ± 0.04^*∗*^	1450.08 ± 337.06	1726.42 ± 217.11	1750.35 ± 215.20	3562.14 ± 427.53	4.68 ± 0.24	5.03 ± 0.33
	Yin-deficiency	2.90 ± 0.65	0.24 ± 0.03	1752.12 ± 321.11^*∗∗*^	3764.53 ± 842.38^*∗∗*^	3782.41 ± 832.52^*∗∗*^	1700.88 ± 406.17^*∗∗*^	5.08 ± 0.74^*∗*^	5.21 ± 0.78^*∗*^

Aucubin	Normal	5.97 ± 2.89	0.38 ± 0.14^*∗∗*^	84.43 ± 32.22	170.50 ± 64.82	204.69 ± 68.61	1358.04 ± 638.52	5.38 ± 2.01	8.58 ± 2.71
	Osteoporosis	3.67 ± 0.78	0.17 ± 0.00	102.55 ± 23.62	116.40 ± 11.00	155.64 ± 24.54	1592.72 ± 276.68	3.06 ± 0.12	5.62 ± 1.09
	Yang-deficiency	7.40 ± 1.68^*∗∗*^	0.25 ± 0.00^*∗*^	95.99 ± 24.19	48.33 ± 14.05^*∗∗*^	111.33 ± 29.94	2328.79 ± 701.23^*∗*^	2.95 ± 0.38	11.69 ± 1.31^*∗∗*^
	Yin-deficiency	8.68 ± 2.92^*∗∗*^	0.25 ± 0.00^*∗*^	181.24 ± 30.66^*∗∗*^	201.40 ± 32.43^*∗∗*^	272.67 ± 70.63^*∗∗*^	959.16 ± 279.31^*∗*^	7.32 ± 1.92^*∗*^	14.14 ± 5.85

Protocatechuic aldehyde	Normal	10.87 ± 4.48	0.45 ± 0.10	18.86 ± 3.62^*∗∗*^	64.15 ± 10.30^*∗∗*^	119.88 ± 32.93^*∗∗*^	750.08 ± 184.83^*∗*^	5.14 ± 0.17^*∗*^	15.85 ± 6.12
	Osteoporosis	12.01 ± 4.69	0.30 ± 0.00	10.10 ± 1.99	34.92 ± 2.37	72.28 ± 21.64	1279.06 ± 400.34	5.52 ± 0.21	17.76 ± 6.26
	Yang-deficiency	2.09 ± 0.89^*∗*^	0.25 ± 0.07	11.38 ± 1.31	24.70 ± 1.69^*∗∗*^	28.68 ± 6.49^*∗∗*^	3031.25 ± 535.72^*∗∗*^	2.55 ± 0.18^*∗∗*^	3.50 ± 1.38^*∗*^
	Yin-deficiency	2.20 ± 0.83^*∗*^	0.17 ± 0.07	8.81 ± 1.61	27.48 ± 3.10^*∗∗*^	28.87 ± 2.69^*∗∗*^	2933.07 ± 265.58^*∗∗*^	3.24 ± 0.37^*∗∗*^	3.79 ± 0.79^*∗*^

Integrated	Normal	4.51 ± 0.44^*∗∗*^	0.25 ± 0.00	11927.33 ± 1782.67^*∗∗*^	17749.62 ± 3510.27	18091.33 ± 3477.17	390.40 ± 78.84	4.57 ± 0.47	5.09 ± 0.40
	Osteoporosis	2.74 ± 0.95	0.25 ± 0.00	7474.98 ± 1002.61	16439.99 ± 6012.37	16516.89 ± 6056.71	472.07 ± 192.41	4.77 ± 1.27	4.87 ± 1.32
	Yang-deficiency	3.91 ± 0.65^*∗∗*^	0.19 ± 0.04^*∗*^	1210.34 ± 269.75^*∗∗*^	1486.54 ± 179.09^*∗∗*^	1507.82 ± 177.63^*∗∗*^	3452.60 ± 389.82^*∗∗*^	4.51 ± 0.23	4.87 ± 0.33
	Yin-deficiency	3.79 ± 0.64^*∗*^	0.25 ± 0.00	14669.30 ± 2504.17^*∗∗*^	38171.03 ± 5606.50^*∗∗*^	38810.41 ± 5480.02^*∗∗*^	184.70 ± 32.39	6.39 ± 0.89	6.80 ± 0.94

^*∗*^ *p* < 0.05 and ^*∗∗*^*p* < 0.01, according to a one-way ANOVA, versus osteoporosis rats.

## Data Availability

The data used to support the findings of this study are available from the corresponding author upon request.
